# COVID-19-Associated Case Fatality Rate in Subjects Under 18 Years Old in Mexico, up to December 31, 2020

**DOI:** 10.3389/fped.2021.696425

**Published:** 2021-10-01

**Authors:** Efraín Navarro-Olivos, Nicolás Padilla-Raygoza, Gilberto Flores-Vargas, María de Jesús Gallardo-Luna, Ma Guadalupe León-Verdín, Elia Lara-Lona, Francisco J. Magos-Vázquez, Daniel Alberto Díaz-Martínez

**Affiliations:** ^1^Directorate of Teaching and Research, Institute of Public Health From Guanajuato State, Guanajuato, Mexico; ^2^Department of Research and Technological Development, Directorate of Teaching and Research, Institute of Public Health From Guanajuato State, Guanajuato, Mexico; ^3^Department of Medicine and Nutrition, Division of Health Sciences, Universidad de Guanajuato, León, Mexico; ^4^Department of Health Services, Institute of Public Health From Guanajuato State, Guanajuato, Mexico

**Keywords:** SARS-CoV-2, COVID-19, children, fatality, co-morbidities

## Abstract

**Background:** The emergence of the SARS-CoV-2 and the COVID-19 have become a global health crisis. The infection has been present in all the social sectors. Subjects under 18 years are one of them. The objective was to analyze the case fatality ratio of COVID-19 cases in the Mexican population under 18 years of age registered in the National Epidemiological Surveillance System from March 2020 to December 31, 2020.

**Material and Methods:** The design is cross-sectional, quantitative, and analytical. All the suspected cases of respiratory viral disease, with a real-time polymerase chain reaction (RT-PCR) test result, aged from 0 to 17 years, were included. Descriptive statistics are presented for all the variables. Epidemiological curves were designed. The chi-squared test and its *P*-values were obtained to show the relationship between comorbidities and death. The case fatality ratio was computed for each comorbidity, sex, and age group. Multivariable logistic regression models were fitted to study the effect between comorbidities with the fatality of cases, adjusting for sex and age group as potential confounders. The alpha value was fixed to 0.05 to assess significance.

**Results:** The number of records for this study was 167,856. Among them, 48,505 were from SARS-CoV-2-positive patients (28.90%), and 119,351 (71.10%) were negative. Of those who died, males (55.29%) (*P* < 0.05) and those under 2 years of age (50.35%) (*P* < 0.05) predominated. Unlike in older populations, from the comorbidities considered risk factors for death by COVID-19, only immunosuppression showed a statistically significant effect on the fatality of cases after adjustment by the other related variables. Sex and age group were not confounders for the models in those under 18 years old. Pneumonia, being younger than 5 years, and immunosuppression are related to death.

**Conclusion:** The case fatality ratio in those under 18 years old is low. Special attention must be paid to those children under 5 years. The development of pneumonia is a warning indicator while treating them. On the other hand, having an open database of cases allows the researchers to analyze the impact of COVID-19 in different population sectors, which has clear benefits for public health.

## Introduction

The emergence of the SARS-CoV-2 and the COVID-19 have become a global health crisis. Differences in the severity of COVID-19 according to the age of patients are reported throughout the literature. Severe disease manifestation, hospitalization need, and mortality risk increase with older age. In contrast, mild COVID-19 is common among child patients (1–3).

Children have been considered less affected than the adult population since they represent 1–5% of the overall infected subjects (3). The cause of differences in lethality by COVID-19 between children and adults could be age-related variations in angiotensin-converting enzyme (ACE)-2 receptor expression (4). The low burden of COVID-19 in children has been seen by many as surprising (5) throughout the world. The effects of the coronavirus disease on children and adolescents depend on family context experiences, which are different within each family and each region in a country (6).

Antúnez-Montes et al. (7), in a study in Latin American children, report that the more frequent comorbidities in severe COVID-19 were congenital heart disease, congenital syndromes, immunological diseases, and neurological disorders. These comorbidities are not included in the open database of the National Epidemiological Surveillance System (NESS) from the General Directorate of Epidemiology (GDE) of the Ministry of Health (8).

In Mexico, among subjects under 18 years old, the first registered COVID-19 case was on March 7, and the first death on April 13, both in 2020 (8). The proportion of COVID-19 cases in this age group is considerably low. As shown by Padilla et al. (9), from 226,089 confirmed cases, 7,040 (3.11%) of them were under 19 years old on June 30, 2020.

Despite a notable increment in confirmed cases and deaths from COVID-19 in Mexico, the population under 18 years old has been the less affected. As reported by Padilla et al. (10), up to July 31, 2020, there were 424,637 confirmed cases, with 14,369 (3.38%) of them under 19 years old, with a cumulative number of 184 deaths in this age group (0.39%).

Few studies analyze the COVID-19-related fatality among subjects under 18 years old in Mexico at the national and the state level. Therefore, the objective was to analyze the case fatality ratio of COVID-19 cases in population under 18 years of age declared to the NESS of the GDE from March 2020 to December 31, 2020 (8).

The research protocol for this study was approved by the Bioethics Committee of Campus Celaya-Salvatierra, University of Guanajuato, Mexico, with the registry CBCCS-05130042020.

## Materials and Methods

### Study Design

This study has a quantitative, cross-sectional, and analytical design.

### Population

We considered all the registries in the NESS database from the Ministry of Health, with all suspected, non-confirmed, and confirmed cases of COVID-19, from March 2020 to December 31, 2020 (8). It is worth noting that in Mexico, all suspected cases of viral respiratory disease are registered in the open database of the NESS (8).

According to the operational definition, a suspected case of viral respiratory disease is anyone of any age with cough, fever, dyspnea (serious), or headache, who also has at least one of the following: myalgias, arthralgias, odynophagia, chills, chest pain, rhinorrhea, anosmia, dysgeusia, and conjunctivitis; in children under 5 years old, irritability is interchangeable with headache (11). If these criteria are met, a real-time polymerase chain reaction (RT-PCR) test or detection of antigen of SARS-CoV-2 is conducted (12). A confirmed case is one in which the test result is positive; meanwhile, for the non-confirmed case, the test result is negative.

### Selection of Participants

The inclusion criteria were all suspected cases of respiratory viral disease, with an RT-PCR test or detection of antigen of SARS-Cov-2 result, aged from 0 to 17 years. The database was examined, and the registries without an RT-PCR test result or detection of antigen of SARS-CoV-2 were deleted, because they remained just as suspected cases, without confirmation nor non-confirmation of the SARS-CoV-2 virus presence.

### Variables

The collected variables in the database were age, sex, Mexican state of registry, date of registry, onset of symptoms date, date of death (if it occurred), result of RT-PCR or antigen of SARS-CoV-2, comorbidities [diabetes, chronic obstructive pulmonary disease (COPD), asthma, hypertension, cardiovascular disease, immunosuppression, chronic kidney disease, obesity, and smoking], pneumonia, and type of patient: treated in hospital or ambulatory. The NESS database did not include multisystem inflammatory syndrome and COVID-19 (MIS-C).

We will be using the following definition of a COVID-19 case: a COVID-19 case is a patient under 18 years with cough, fever, dyspnea (serious), or headache (in patients under 5 years it can be interchangeable with irritability), accompanied with myalgias, arthralgias, shore throat, chills, thoracic pain, rhinorrhea, anosmia, dysgeusia, and conjunctivitis, with a RT-PCR test result positive for SARS-CoV-2 or positive detection of SARS-CoV-2 antigen (11, 12).

Mexican states were grouped according to the regions defined by the National Institute of Public Health in the National Survey of Health and Nutrition (13): north: Baja California, Baja California Sur, Chihuahua, Coahuila, Nuevo León, Sinaloa, Sonora, and Tamaulipas; center: State of Mexico, Mexico City, Hidalgo, Morelos, Puebla, Querétaro, and Tlaxcala; western-center: Aguascalientes, Colima, Durango, Guanajuato, Jalisco, Michoacán, Nayarit, San Luis Potosí, and Zacatecas; and South-Southeast: Campeche, Chiapas, Guerrero, Oaxaca, Quintana Roo, Tabasco, Veracruz, and Yucatán.

### Statistical Analysis

For all the variables, descriptive statistics are presented. Epidemiological curves were designed for confirmed cases of COVID-19 by date of onset of symptoms and another one corresponding to deaths due to COVID-19 by date of death. The chi-squared test and the computation of its *P*-value were conducted to show differences between confirmed and non-confirmed cases. The case fatality ratio (CFR) was computed for each Mexican state. The chi-squared test was used to assess the relationship between comorbidities and death from COVID-19, presenting its respective *p*-value and degrees of freedom. A logistic regression model was fitted for each underlying pathology and death, adjusting for sex and age group as potential confounders [reporting the odds ratio (OR) and their confidence intervals at 95% level (95% CI)]. All comorbidities that showed a relationship with death were included in a multivariate logistic regression model to obtain an OR and 95% CI, adjusted for the rest of the comorbidities. The alpha value was fixed to 0.05 to show statistical significance. The statistical analysis was performed in Stata 13.0® (Stata Corp., College Station, TX, USA).

## Results

The total number of records corresponding to subjects under 18 years old was 167,856. Among them, 48,505 (28.90%) tested positive for SARS-CoV-2, and 119,351 (71.10%) tested negative for SARS-CoV-2. Among the confirmed cases, 24,459 (50.43%) were males and 24,046 (49.57%) were females.

The distribution of confirmed cases by age groups was as follow: 5,573 (11.49%) were from 0 to 2 years, 3,666 (7.56%) from 3 to 5 years, 11,696 (24.11%) from 6 to 11 years, and 27,570 (56.84%) from 12 to 17 years.

**Figure 1:**
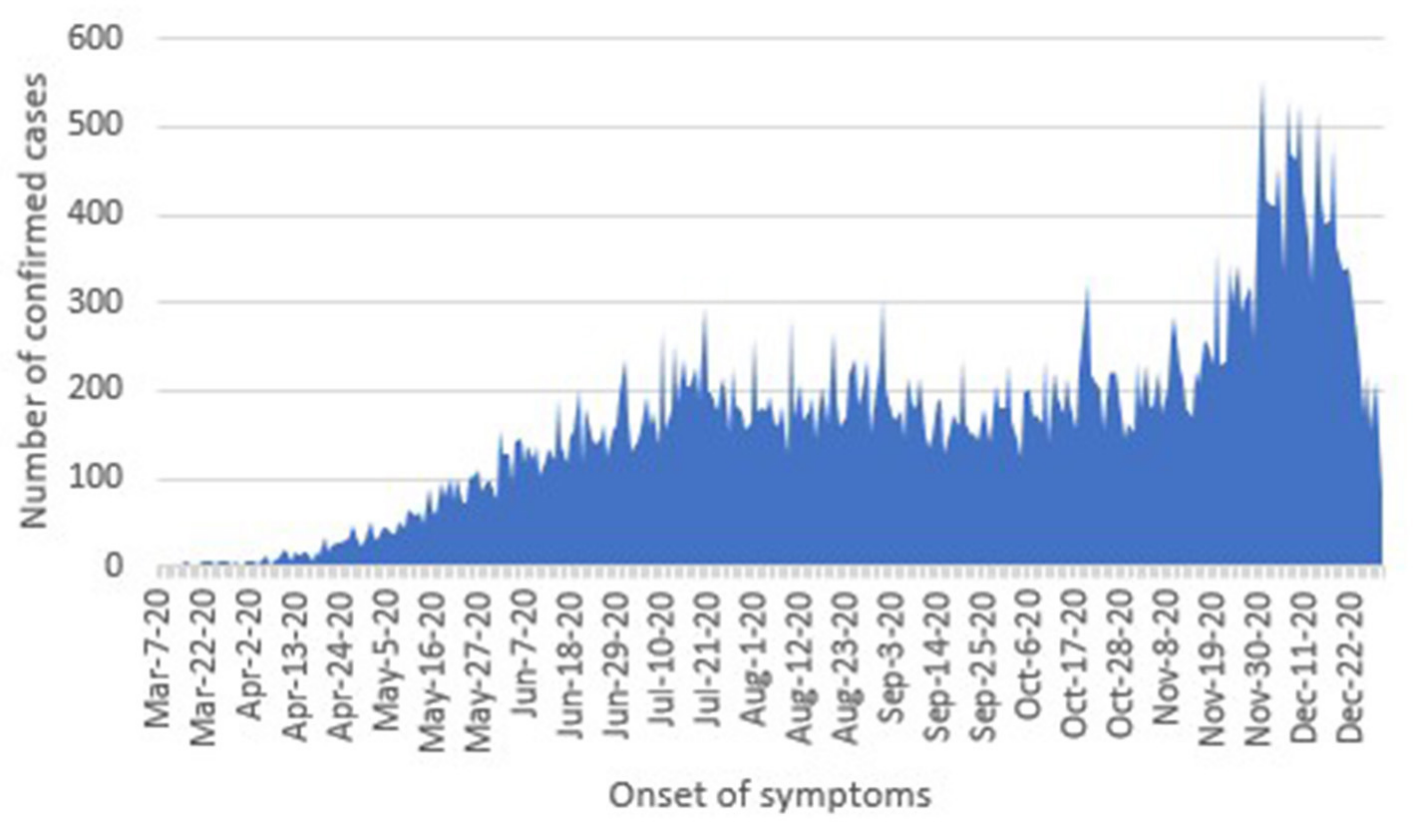
Distribution of confirmed cases by onset of symptoms. Source: GDE (8).

From March to mid-November 2020, the number of daily cases was around 200 with some increments. Nevertheless, in the third week of November and throughout December, the number of new confirmed cases increased ~2-fold (Figure 1).

**Figure 2:**
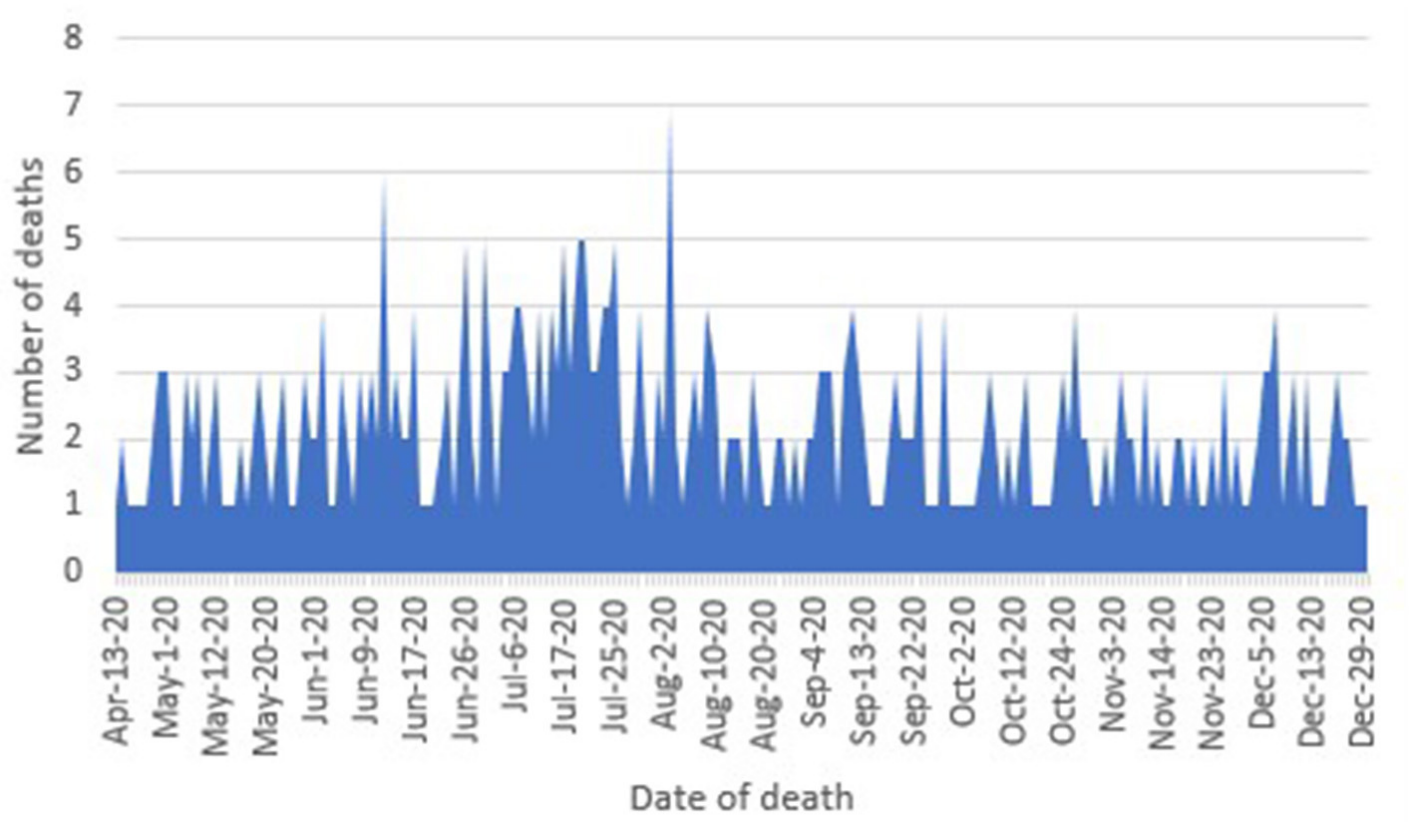
Curve by date of death in COVID-19 confirmed cases. Source: General Directorate of Epidemiology (8).

From June to August 2020, the number of deaths in the population under 18 years old increased in Mexico; between September and December, this number decreased with some localized peaks (Figure 2).

The number of men was higher than the number of women among those who died from COVID-19, showing a relationship between sex and death (*P* < 0.05), which is also reflected with a higher CFR among men than in women. By age group, those under 2 years of age predominated among those who died from the infection by SARS-CoV-2. A relationship between age group and death from COVID-19 is shown (*P* < 0.05), with a CFR of 3.99% for those between 0 and 2 years old, being the highest CFR among the age groups. Concerning Mexican regions, those who resided in the center predominated, showing a relationship between region and death due to COVID-19 (*P* < 0.05). The highest CFR was 1.60%, which corresponded to the South-Southeast of Mexico. At the national level, the CFR was 0.88% in those under 18 years of age (Table 1).

**Table 1:** Distribution of case fatality ratio, by sex, age group, and area in Mexico.

**Variables**	**Confirmed cases *n***	**Number of deaths *n***	**Case fatality ratio % (95% CI)**
Sex	*X*^2^ = 4.07; DF 1	*P* = 0.04	
Male	24,224	235	0.96 (0.84–1.09)
Female	23,856	190	0.80 (0.69–0.92)
Age group (years)	*X*^2^ = 639.64; DF 3	*P* < 0.05	
0–2	5,359	214	3.99 (3.48–4.55)
3–5	3,640	26	0.71 (0.47–1.04)
6–11	11,634	62	0.53 (0.41–0.68)
12–17	27,447	123	0.45 (0.37–0.53)
Mexican areas	*X*^2^ = 83.31; DF 3	*P* < 0.05	
North	8,334	109	1.31 (1.08–1.58)
Center	25,565	141	0.55 (0.46–0.65)
West-center	8,593	79	0.92 (0.73–1.14)
South-southeast	6,013	96	1.60 (1.30–1.95)
National	48,505	425	0.88 (0.80–0.96)

*DF, degree of freedom*.

*Source: General Directorate of Epidemiology (8)*.

Female sex had a protective effect on death by COVID-19 (OR 0.82; 95% CI 0.68–0.99). Nevertheless, this effect was confounded by age group and presence of asthma and immunosuppression (Table 2). The effect of the age group on death by COVID-19 was stronger in those under 2 years, followed by the corresponding effect on the age group of 3–5 years. For the other age groups (6–11 and 12–17 years), the OR obtained were not statistically significantly distinct from one (Table 2).

**Table 2:** Relation between comorbidities and death among COVID-19 confirmed cases.

	**Deaths *n* (%)**	**Non-deaths*n* (%)**	**OR univariate (95% CI)**	***P*-value**	**OR multivariable (95% CI)**	***P*-value**
Sex			0.82 (0.68–0.99)	0.04	0.86 (0.71–1.04)	0.13
Female	190 (44.71)	23,856 (49.62)				
Male	235 (55.29)	24,224 (50.38)				
Age group (years)						
0–2	214 (50.35)	5,359 (12.14)	10.32 (8.21–12.97)	<0.05	10.14 (8.06–12.74)	<0.05
3–5	26 (6.12)	3,640 (7.57)	2.14 (1.52–3.00)	<0.05	2.12 (1.51–2.98)	<0.05
6–11	62 (14.59)	11,634 (24.20)	1.19 (0.88–1.61)	0.27	1.19 (0.87–1.61)	0.28
12–17	123 (28.94)	27,447 (57.09)	–	–		
Diabetes	*X*^2^ = 265.02	DF 1; *P* < 0.05	1.01 (0.99–1.03)	0.31	–	–
Yes	32 (7.55)	332 (0.69)				
No	392 (92.45)	47,688 (99.31)				
COPD	*X*^2^ = 1.10	DF 1; *P* = 0.6	0.96 (0.73–1.24)	0.74	–	–
Yes	1	52			–	
No	424	47,974				
Asthma	*X*^2^ = 6.16	DF 1; *P* = 0.05	0.36 (0.15–0.87)	0.02	0.48 (0.20–1.18)	0.11
Yes	5	1,544				
No	420	46.480				
Immunosuppression	*X*^2^ = 252.60	DF1; *P* < 0.05	1.01 (0.99–1.03)	0.26	1.03 (1.01–1.18)	0.002
Yes	40	527				
No	384	47,495				
Hypertension	*X*^2^ = 178.27	DF1; *P* < 0.05	0.99 (0.95–1.04)	0.72	–	–
Yes	24	274				
No	401	47,753				
Cardiovascular disease	*X*^2^ = 109.93	DF 1; *P* < 0.05	0.99 (0.94–1.04)	0.67	–	–
Yes	21	322				
No	404	47,701				
Chronic kidney disease	*X*^2^ = 304.72	DF1; *P* < 0.05	0.99 (0.95–1.04)	0.71	–	–
Yes	23	152				
No	402	47, 873				
Obesity (≥30 kg/m^2^)	*X*^2^ = 12.39	DF1; *P* = 0.002	0.99 (0.94–1.04)	0.66	–	–
Yes	30	1,836				
No	395	46,192				
Smoking	*X*^2^ = 2.65	DF 1; *P* = 0.27	0.97 (0.87–1.09)	0.62	–	–
Yes	7	459				
No	418	47,561				

*OR, odds ratio; COPD, chronic obstructive pulmonary disease; DF, degree of freedom*.

*Source: General Directorate of Epidemiology (8)*.

Unlike in older populations, the comorbidities considered risk factors for death do not show a statistically significant effect on the fatality of cases (taking the univariate logistic regression model).

According to the chi-squared test, the comorbidities related to death were diabetes, asthma, immunosuppression, hypertension, chronic kidney disease, and obesity. Nevertheless, a logistic regression model for each variable as the unique predictor for death shows that only age group (0–2 and 3–5 years), immunosuppression, and asthma have a statistically significant effect (Table 2).

A multivariate logistic regression model was fitted with these variables. From the considered variables, only age group (0–2 and 3–5 years) and immunosuppression had a statistically significant effect on the fatality of COVID-19 cases (Table 2).

The CFR of pneumonia was 15.46% in the Mexican population under 18 years old. The effect of pneumonia on death by COVID-19 is strong (OR = 63.90; 95% CI 51.75–78.92); this effect was not confounded by sex (OR = 63.37; 95% CI 51.75–78.92), but it was confounded by age group (OR = 46.62; 95% CI 37.35–58.20).

## Discussion

In Mexico, up to December 31, 2020, 1,426,094 confirmed cases of COVID-19 were reported in the GDE database (8), and the cumulative number of confirmed cases in subjects under 18 years old was 48,505, which represents 3.40% of overall cases. It verifies that the presence of SARS-CoV-2 and COVID-19 is less frequent in pediatric ages than in adults.

In the early months of the COVID-19 pandemic, the prevalence in children was extremely low, as reported in Huang et al. (14), where among 41 studied cases, none were under 18 years old. Also, it was reported in Padilla et al. (9) that by June 30, 2020, in Mexico, there were 7,040 (3.12%) cases among subjects under 19 years old. Another study, made by The Chinese Novel Coronavirus Pneumonia Emergency Response Epidemiology Team, studied 72,314 subjects, reporting that only 2.1% were persons between 0 and 19 years old (15), being less than that reported in Mexico of 3.4% (8).

The highest number of cases reported by Dong et al. (16) were at younger ages, unlike in Mexico, where children from 12 to 17 years old predominated. Both in Dong et al. (16) and the present study, there was no predominance of sex (Table 1).

Rivera-Hernández et al. (17), using the NESS database up to October 19, 2020, reported the characteristics of COVID-19 cases grouped by 0–14, 15–64, and 65+ years. It is shown that among the first group (0–14), all the comorbidities registered in the database have a low prevalence in accordance with this study (Table 2).

Regarding China, in subjects from 10 to 19, the CFR was 0.2%, and there were no deaths among those under 10 (15). By contrast, in Latin-America, Antúnez-Montes et al. (7), reported a CFR of 4.16%. In Mexico, at the national level, the CFR for those under 18 years was 0.88% (Table 1). The observed CFR among children is low for the Mexican population. The authors believe this is due to the following reasons: biological ones such as the variations in angiotensin-converting enzyme (ACE)-2 receptor expression (whose verification is out of the scope of this study) and timely health care access for those who present complications such as pneumonia. The CFR among those with pneumonia was nearly 15.46%, and the OR was above 40. It shows the importance of this complication and its prevention.

Lu et al. (18) report cases of COVID-19 in children and their comorbidities (that do not correspond to the comorbidities in adults, i.e., hydronephrosis, leukemia, and intussusception) that represent a high effect on mortality. Antúnez-Montes et al. (7) found that those with severe COVID-19 had comorbidities, including congenital heart disease, congenital syndromes, immunological diseases, and neurological disorders. The registry in Mexico (8) did not include sufficient information for differentiating those diseases [neither the ones mentioned by Lu et al. (18) nor by Antúnez-Montes et al. (7)], and so the comparison with these studies is not straightforward. The comorbidities that are considered a risk factor for dying from COVID-19 included in the NESS database do not show any effect on fatality except immunosuppression in the Mexican population under 18 years (Table 2). It is explained because diabetes, hypertension, cardiovascular disease, chronic kidney disease, COPD, and others are more prevalent in the adult population.

In the multivariable logistic regression model, sex does not show a statistically significant effect on death by COVID-19. For the age groups, those under 5 years have a statistically significant OR distinct to one. It is worth noting the high value for the age group 0–2 years. For the comorbidities included, only immunosuppression showed a statistically significant effect.

As complications due to COVID-19, it is reported that 64.9% of children with COVID-19 developed pneumonia (15). In Mexico, 1,889 children under 18 years of age (3.89%) had a diagnosis of pneumonia, of which 292 (15.46%) died. Rivera-Hernández et al. (17) reported a higher pneumonia diagnosis in the Mexican population under 15 years (6.1%), until October 2020.

Historically, Mexican states from the south have a high marginalization index compared to the states from the center and north. It is worth noting the variability of the CFR by regions (Table 1). It may be due to various factors, including the timely and quality of attention. For future work, a sociodemographic analysis as a factor for the fatality of cases is of interest.

Among children, 13% of confirmed cases of SARS-CoV-2 infection have been reported as asymptomatic (19). In Mexico, according to the suspected case of viral disease operational definition (11), only the ones presenting symptoms were subjected to RT-PCR testing. Therefore, we cannot quantify asymptomatic children in this study, which may be a limitation and a bias.

It was hypothesized in previous analyses that the low rate of infection in children was due to the mobility restrictions imposed in the early phase of the pandemic (16). Nevertheless, this hypothesis does not sound plausible in the light of this and other studies (1–3, 19), given the time that passed from the first infections.

While assessing the risk factors of reopening social activities and planning, COVID-19 in children must be understood adequately. They represent a social sector highly exposed to interactions with other persons due to their academic activities, which shows the importance of this kind of analysis.

The fitted multivariate logistic regression model did not show a statistically significant effect of the comorbidities on death by COVID-19 (Table 2), except for immunosuppression, contrasting with older age groups, which present significant effects on lethality (9, 10). Also, asthma lost its protective effect after adjusting by comorbidities and sex. The same model shows a strong effect on the lethality of COVID-19 of the age group for those under 2 years (Table 2).

### Strengths

The sample size for this study is large, which impacts positively in the statistical precision of the results. The individual-level resolution of the NESS database allows us to conduct a more detailed statistical analysis, which included different multivariable logistic models. A detailed registry enables the impact analysis of COVID-19 in the Mexican population under 18 years of age and other specific groups.

### Limitations

Due to the origin of the data, a surveillance system, the scope of this study is limited. The principal bias is that the database consulted is designed for epidemiological surveillance of the general population. Hence, a limitation of the study is that the database used, to date, does not include differentiation of congenital heart disease, congenital disorders, and immunological disorders that, as an example, Antúnez-Montes et al. (7) reported as related with severe COVID-19. Nevertheless, it has been shown that the CFR was higher among those under 2 years old than in the other age groups considered (Table 2). The study of this phenomenon deserves a closer view.

The NESS database does not report MIS-C.

## Conclusion

The CFR in those under 18 years old is low compared with the one corresponding to those aged 18 and older. The main condition related to death by COVID-19 in the population under 18 years old in Mexico is pneumonia. The comorbidities considered in the GDE database do not show an association with death in these age groups, except immunosuppression.

The CFR is high in southeast Mexican states (Chiapas, Morelos, Veracruz, Guerrero, Tlaxcala, Quintana Roo, and Oaxaca), besides some in the north (Aguascalientes and Baja California).

Sex did not play a statistically significant role in death by COVID-19 for the children population considered. Those under 5 years are more prone to death than the older ones. Those in the group 0–2 years showed the highest odds ratio for death by COVID-19.

Special attention must be paid to those children under 5 years. The development of pneumonia is a warning indicator while treating them. On the other hand, having an open database of cases allows the researchers to analyze the impact of COVID-19 in different population sectors, which has clear benefits for public health.

## Data Availability Statement

The datasets obtained from General Directorate of Epidemiology for this study can be found in Open Science Framework [Padilla-Raygoza N. Fatality of COVID-19 in children Dec 31, 2020 (2020). Availabe in: https://osf.io/6xb28/].

## Ethics Statement

The studies involving human participants were reviewed and approved by Bioethics Committee, Campus Celaya-Salvatierra, University of Guanajuato. Written informed consent from the participants' legal guardian/next of kin was not required to participate in this study in accordance with the national legislation and the institutional requirements.

## Author Contributions

EN-O designed the protocol, analyzed the data, and wrote the first draft of the manuscript. NP-R participated in designing the protocol, analyzing the data, and participated in writing the first draft of the manuscript. GF-V participated in clearing the database and participated in the analysis of the data. MG-L obtained the database, checked that registries had all data, and participated in writing the first draft of the manuscript. ML-V participated in the analysis of data and writing the first draft. EL-L searched the literature and made a critical review of the protocol and the first draft of the manuscript. FM-V had the idea and participated in the analysis of data and writing the first draft of the manuscript. DD-M participated in the review of the protocol and in writing the first draft of the manuscript. All authors contributed to the article and approved the submitted version.

## Funding

The University Autonomous of Guadalajara will support the fee for publishing the manuscript.

## Conflict of Interest

The authors declare that the research was conducted in the absence of any commercial or financial relationships that could be construed as a potential conflict of interest.

## Publisher's Note

All claims expressed in this article are solely those of the authors and do not necessarily represent those of their affiliated organizations, or those of the publisher, the editors and the reviewers. Any product that may be evaluated in this article, or claim that may be made by its manufacturer, is not guaranteed or endorsed by the publisher.
